# Dysphagia rehabilitation following acquired brain injury, including cerebral palsy, across the lifespan: a scoping review protocol

**DOI:** 10.1186/s13643-021-01861-9

**Published:** 2021-12-13

**Authors:** Rhiannon Halfpenny, Alexandra Stewart, Paula Kelly, Eleanor Conway, Christina Smith

**Affiliations:** 1 Great Ormond Street Hospital, Great Ormond Street, London, UK; 2 University College London, London, UK

**Keywords:** Dysphagia, Rehabilitation, Acquired brain injury (ABI), Cerebral palsy (CP), Children, Adults

## Abstract

**Background:**

Swallowing impairment (dysphagia) following brain injury can lead to life-threatening complications such as dehydration, aspiration pneumonia and acute choking episodes. In adult therapeutic practice, there is research and clinical evidence to support the use of swallowing exercises to improve swallowing physiology in dysphagia; however, the use of these exercises in treating children with dysphagia is largely unexplored. Fundamental questions remain regarding the feasibility and effectiveness of using swallowing exercises with children. This review aims to outline the published literature on exercise-based treatment methods used in the rehabilitation of dysphagia secondary to an acquired brain injury across the lifespan. This will allow the range and effects of interventions utilised to be mapped alongside differential practices between adult and child populations to be formally documented, providing the potential for discussions with clinicians about which rehabilitative interventions might be appropriate for further trial in paediatrics.

**Methods:**

This study will use a scoping review framework to identify and systematically review the existing literature using Joanna Briggs Institute (JBI) and Preferred Reporting Items for Systematic Reviews (PRISMA) scoping review guidelines. Electronic databases (MEDLINE, PubMed, Cumulative Index to Nursing and Allied Health Literature (CINAHL) and Allied and Complementary Medicine Database (AMED)), grey literature and the reference lists of key texts including systematic reviews will be searched. Information about the rehabilitation design, dosage and intensity of exercise programmes used as well as demographic information such as the age of participants and aetiology of dysphagia will be extracted. The number of articles in each area and the type of data source will be presented in a written and visual format. Comparison between the literature in adult and child populations will be discussed.

**Discussion:**

This review is unique as it directly compares dysphagia rehabilitation in adults with that of a paediatric population in order to formally identify and discuss the therapeutic gaps in child dysphagia rehabilitation. The results will inform the next stage of research, looking into the current UK-based speech and language therapy practices when working with children with acquired dysphagia.

**Systematic review registration:**

Open science framework osf.io/ja4dr

**Supplementary Information:**

The online version contains supplementary material available at 10.1186/s13643-021-01861-9.

## Introduction

Acquired brain injury (ABI) is an umbrella term used to describe the damage that occurs to the brain after birth that is not associated with a hereditary or progressive disease. It can be characterised by traumatic brain injury (TBI) and non-traumatic brain injury (nTBI). Traumatic brain injury refers to damage to the brain from an external force such as a blunt force object, car accident or fall. In contrast, nTBI arises from internal damage to the brain such as stroke, a brain tumour or asphyxiation.

The incidence of ABI can vary across the lifespan. It is estimated that around 348,934 patients per year are admitted to the hospital with an acquired brain injury [1] with roughly 40,000 of these cases occur in children [2]. In older adults, ABI is typically linked to cerebrovascular accidents such as stroke whereas in teenagers and younger adults, traumatic brain injuries from external trauma and car accidents are more common [3]. Acquired brain injury in infants and young children typically arises from a range of causes including birth trauma, brain tumours and infections [4]. Children sustaining a brain injury in early life, before the age of 2 years, may go on to receive a diagnosis of cerebral palsy (CP) [5].

The management of ABI also differs across the lifespan. Children are far more likely to be discharged to their home environment following a brain injury than adults [6] and typically have less access to specialist therapeutic services to support the rehabilitation of a wide range of morbidities which can occur following ABI [7].

Dysphagia (swallowing difficulty) is one of these possible morbidities, with studies recording dysphagia in up to 93% of people with ABI [8]. Although the severity of dysphagia varies, dysphagia in any form can cause psychological and physical consequences such as anxiety, embarrassment, social isolation and increased risks of pneumonia, dehydration and mortality [9, 10]. Weight loss and poor nutrition are other possible complications of dysphagia, especially due to the increased metabolic demands placed on the body following a brain injury [11]. This is especially pertinent in children, where poor nutrition can lead to faltering growth, impacting the overall physical and cognitive development [12].

Typically, management of dysphagia, in both adults and children, has focused on increasing the safety of swallowing via indirect strategies such as thickened fluids, positional support or supplementary feeding methods such as percutaneous endoscopic gastrostomy (PEG). Although these management strategies aim to reduce the risk of aspiration pneumonia, they do not change the underlying swallowing function and do little to combat the psychosocial isolation someone with dysphagia may experience. Eating and drinking often form a significant emotional and social part of someone’s everyday life, and texture modification or supplementary tube feeding can significantly impact this social participation. The need to consider direct rehabilitation options in order to improve the swallowing physiology is therefore vital in order to make life-changing medical, psychosocial and economic differences.

In the 1980’s direct rehabilitation strategies to restore the physiological functioning of swallowing in adult populations emerged [13]. Initial approaches used sensory-based stimulation methods such as ‘thermal tactile stimulation’ which involves stimulating the anterior faucial pillars of the oral cavity with a cold probe. The aim of this is to increase the sensitivity of the oral cavity and therefore stimulate a timely swallow trigger [14]. Rehabilitation then progressed onto using specific exercises to target weak oro-pharyngeal musculature, for example, the ‘effortful swallow’ was used to improve contact between the base of the tongue and posterior pharyngeal wall [15], the ‘Mendelson manoeuvre’ to improve laryngeal elevation [16] and the ‘head lift’ to improve hyoid displacement [17]. More recently, rehabilitation has focussed on re-acquiring the ‘skill’ of swallowing through specific exercise programmes [18]. Skill in this context refers to an ability to regulate the precision and timing of swallowing in relation to a bolus.

Strategies to improve patient understanding, engagement and performance when performing these exercises have since been introduced. These strategies often use electronic devices in order to provide online biofeedback regarding the accuracy of the exercises being performed. An example of a biofeedback system in adult rehabilitation practice is ‘surface electromyography’ (sEMG) [19]. This measures the timing and force of muscle contraction using electrodes placed on a selected area and provides a visual, graphical representation of those measures. The visual feedback can act as a reference point during therapy for patients to measure their performance. Another area of development involving technology is neuromuscular electrical stimulation (NMES). This can be used in isolation or combined with exercises to electrically stimulate the oropharyngeal muscles. Electrodes are placed at specific points around the lower jaw and neck to stimulate the targeted pharyngeal musculature and strengthen the directed muscles. There is evidence to suggest that combining oro-pharyngeal exercises with biofeedback and/or electrical stimulation increases motivation, improves the accuracy of movement and generates better functional outcomes for patients [20–23]. Despite the therapeutic advances in adult populations described above, there remains a generalised lack of research into the physical, cognitive and emotional rehabilitation of children post-brain injury [6]. In dysphagia practice, young people continue to rely on indirect, conservative feeding strategies to manage their dysphagia [24]. Expert opinion guidelines for the management of paediatric acquired brain injury still recommend the use of rehabilitative swallowing exercises from a theoretical perspective but recognise the need for specific research in this area [25]. One possible reason for this is that developing therapeutic protocols in paediatric populations is more challenging given the overall incidence of ABI is smaller. Whilst it is not always possible to make direct translations from approaches used in adult populations to that of paediatrics [26], there is evidence in other therapeutic areas of developing paediatric interventions from the adult literature. For example, the use of ‘functional electrical stimulation’ in upper limb therapy has been applied in the treatment of children with CP based on research from the adult ABI population [27]. Having a clear understanding of the scope and effectiveness of rehabilitative interventions described in both the adult and paediatric literature is therefore a key first step in developing the evidence base in paediatrics.

The aim of this paper, therefore, is to outline the published literature on exercise-based treatment methods used in the rehabilitation of dysphagia secondary to an acquired brain injury across the lifespan. This will be used to identify intervention differences and gaps between adult and paediatric populations and guide discussions with clinicians about what rehabilitative approaches might be applicable for further trials in paediatrics. For the purposes of this study, cerebral palsy is included in the definition of acquired brain injury.

## Developing the research question

Previous clinical guideline papers have highlighted the lack of available literature on the rehabilitation of swallowing following brain injury in children [25]. Experience from clinical practice highlights that this can be a source of frustration for parents and families who frequently ask if there are any rehabilitation strategies to help resolve their child’s swallowing impairment because they may have heard or read about available treatments in adults. It was therefore felt important to include both adult and paediatric literature in this review to enable the research gaps to be formally acknowledged and reported. For the purposes of this study, children with CP are also included under the term ABI as some children with CP will have received this diagnosis following a brain injury in the post-natal period and beyond. As children with CP typically present with a high incidence of dysphagia, we do not want to exclude them from our data collection.

The research question posed by the researchers following on from this decision was *What rehabilitation options are available for people with dysphagia secondary to ABI?* This question was used to conduct a pilot literature search to gain up to date information about treatment methods available in both adult and paediatric populations. The search highlighted that treatment options for dysphagia rehabilitation could be separated into several groups: surgical, pharmaceutical, cortical and peripheral stimulation, alternative therapies and direct oro-pharyngeal exercises. Given the breadth of treatment options, the question was subsequently redefined to explore one of these treatment groups: oro-pharyngeal exercises. The use of exercises in the rehabilitation of paediatric dysphagia is recognised as a research priority by the Royal College of Speech and Language Therapists (RCSLT) and the National Institute of Health Research (NIHR) [28], and their use has also been recommended in paediatric brain injury therapeutic guidelines based on expert consensus opinion [25].

Therefore, the primary research question posed by the researcher is: *What direct oro-pharyngeal exercise protocols are available for adults/children with dysphagia post-acquired brain injury?*

## Method/design

As this study aims to provide an outline of available literature and confirm a suspected therapeutic gap in the literature, it will use a scoping review methodology [29]. A broader, exploratory review of the data is indicated so that the available literature in both adult and paediatric populations can be mapped and compared [30]. This will allow the investigators to review the range of protocols and methodologies utilised by different researchers in a similar area which can be used to guide discussions into rehabilitation options for paediatrics in the future.

This study protocol has been registered within the Open Science Framework (registration number: ja4dr) and is being reported in accordance with the reporting guidance provided in the Preferred Reporting Items for Systematic Reviews and Meta-Analyses Protocols (PRISMA-P) statement [31] and the PRISMA Extension for scoping reviews (PRSIMA-SCR) [32] (see checklist in Additional file 1). This scoping review protocol will be conducted by using the Joanna Briggs Institute (JBI) guidelines for conducting scoping reviews to ensure systematic and repeatable work [33] and will follow the five stages included when conducting a scoping review as outlined by Arksey and O’Malley [34].

### Inclusion criteria

The following are the inclusion criteria:

1. Children and adults of any age.
2. Participants with dysphagia secondary to an acquired brain injury including but not restricted to stroke, traumatic and non-traumatic brain injury, cerebral palsy, brain neoplasm and autoimmune disorders.
3. Direct oro-pharyngeal exercises. These are defined as exercises involving the oro-pharyngeal musculature with the aim of changing participant swallowing physiology. These include, but are not be restricted to, strength-based exercises (e.g. effortful swallow, Mendelsohn manoeuvre), respiratory coordination exercises (e.g. expiratory muscle strength training) and skill-based programmes (e.g. BiSSKiT protocol).
4. Exercise protocols that use external devices as an adjunct to rehabilitation (e.g. biofeedback technology/electrical stimulation/oral appliances).

### Exclusion criteria

The following are the exclusion criteria:

1. Dysphagia arising from other causes such as head and neck cancers, structural abnormalities, idiopathic myopathies and genetic or inherited conditions.
2. Children with primary aversive or sensory behavioural feeding difficulties which prevent them from wanting to eat and drink.
3. Interventions involving pharmaceutical management (e.g. Botox), cortical stimulation (e.g. repetitive transcranial stimulation), peripheral stimulation in isolation (e.g. pharyngeal electrical stimulation) and surgery or alternative treatments (acupuncture) will not be included in this review. There may be scope for analysing these interventions in a future scoping review.
4. Compensatory strategies which do not involve adaptation of the oro-pharyngeal musculature. For example, the chin tuck manoeuvre or texture/taste modifications.
5. External compensatory equipment.
6. Animal studies.

#### Sources of evidence used as eligibility criteria

The breadth of a scoping review means that multiple data sources can be considered, over and above what would typically be included in a systematic review [30]. As a method of ensuring robust data is reviewed, the following inclusion and exclusion criteria will be applied to the type of information sources obtained in this review.

Inclusion:

- Case reports, case series, experimental studies, randomised control trials and observational studies.

Exclusion:

- Commentaries, opinion pieces and systematic reviews will be excluded, but their reference lists will be reviewed for appropriate references that fulfil the outlined criteria.
- Articles not written or available in English.
- Where a full-text article cannot be obtained using university access, the 1st author will be contacted. If no copy is made available prior to the final analysis, then the paper will be excluded.
- Articles will need to report sufficient treatment information including treatment type, dosage and intensity. Articles without sufficient protocol information as outlined will be excluded.
- As this review aims to identify recent evidence, papers dated before 2005 will not be included.

### Databases to be searched

Initial searches will be conducted via the following electronic databases (from their inception onwards): MEDLINE (via EBSCOhost), PubMed (via EBSCOhost), CINAHL Plus (via EBSCOhost), AMED (via OVID) and Cochrane Database of Systematic Reviews (via OVID).

One reviewer will hand-search reference lists of included articles, and relevant articles will be included. Grey literature identified via social media, open access thesis, conference proceeding abstracts, dissertations or from clinical experts in this field will also be considered if they meet the outlined inclusion/exclusion criteria.

### Search strategy

The search strategy has been developed with the Institute of Child Health, University College London librarian. Identified terms will be searched within the Medical Subject Heading (MeSH), followed by a keyword search for each database. Two searches will be run, on each database to ensure no papers including the child population are excluded. Search 1 includes search terms for levels 1, 2 and 3, and this search will be repeated with level 4 search terms added. A record of the number of articles found on each database will be made. A draft search strategy is available in Additional file 2.

### Study selection

One reviewer will run the initial searches and export the titles and abstracts into Rayyan QCRI [35]. Duplicate copies will be deleted. The reviewer will then screen the title and abstracts of each paper for inclusion or exclusion. Two further reviewers will each check 10% of these decisions using the Rayyan online software. Reviewers will be blinded to the decisions. Conflicts and further questions will be discussed and clarified, and a majority decision will be taken if these conflicts cannot be resolved. A record of decisions will be kept on Rayyan QCRI. If there is a disagreement greater than 20%, then a second reviewer will screen all papers.

### Data extraction

Following an initial screening, whole-text articles for the included articles will be sought. The following data will be extracted from each article and collated by the primary researcher: title, methodology, participant demographics, baseline aetiology and outcome measures. Details of the exercise protocol will also be extracted including the type of exercises, dosage, intensity and format. This data will be reviewed and analysed against the outlined inclusion/exclusion criteria by the primary researcher. Two further reviewers will each check 20% of these decisions. A majority decision will be taken if there is a disagreement between reviewers. If there is a disagreement greater than 20% then a second reviewer will screen all papers. Rayyan QCRI online software will be used to record the final decisions.

### Data synthesis

Data will be categorised into articles involving children under the age of 18 years old and adults 18 years and above. Based on the pilot literature review and experience in clinical practice, it is anticipated that certain sub-groups of treatments will be found. These include the following:

1. Exercises in isolation targeting the oral/pharyngeal or respiratory systems used in swallowing
2. Exercises combined with biofeedback
3. Exercises used in conjunction with electrical stimulation

The volume of articles per sub-category related to each age group (adult vs child) will be visually charted using a table and bubble chart. Data relating to specific study characteristics (such as intervention, methodological design, dosage, outcome measures) will be presented in the descriptive numerical form alongside a narrative summary. Comparison between the literature in adult and child populations will be considered and discussed using a narrative review.

Although the specific rigour of each paper will not be discussed in detail, descriptive information about the level of each review paper (e.g. randomised control trial/case report) will be made. The inclusion criteria and protocol design of each paper will also be reviewed in order to determine the possibility of trialling certain methods with a new population. For example, trialling a method used in adult post-stroke dysphagia with children with cerebral palsy.

## Discussion

This scoping review is intended to outline the published literature on exercise-based treatment methods used in the rehabilitation of dysphagia secondary to an acquired brain injury across the lifespan. The results will be used to inform future research studies exploring the use of rehabilitation strategies in children with dysphagia, secondary to an acquired brain injury. Given the apparent lack of rehabilitation studies in paediatric dysphagia populations, it is hoped that by mapping the literature found in both adult and paediatric populations, the similarities and differences between the populations can be discussed in order to confirm a suspected gap in the paediatric literature and guide future research agendas. As this is an exploratory review of the literature, it is recognised that conducting the review might highlight further questions, leading to further refinement of the research question or protocol. Deviations from this protocol will be recorded by the research team and on an open science framework. Changes will be clearly stated alongside their rationale in any future dissemination of results.

Shared access to sources of information among the research team will be made possible via the use of open access screening and selection software (Rayyan QCRI). This is of particular importance during the COVID-19 pandemic as research teams may not be able to meet in person as consistently.

### Limitations

A possible criticism of this protocol is that it only explores exercise-based rehabilitative techniques. There were several reasons behind this decision. Firstly, clinical practice guidelines created by specialists in the field of paediatric brain injury have recommended the use of exercises in swallowing rehabilitation in this population [25]. Secondly, the use of exercises in the rehabilitation of paediatric dysphagia is recognised as a research priority by the Royal College of Speech and Language Therapists (RCSLT) and the National Institute of Health Research (NIHR), 2018 [28]. Finally, although scoping reviews allow for broad data collection, the authors do not want to dilute the outcomes by having an extremely broad subject area. Further reviews exploring the available literature targeting other areas including surgical, pharmaceutical and cortical stimulation can be considered in the future if felt clinically applicable.

As a scoping review protocol and not a systematic review protocol is being utilised, it is anticipated that the results obtained will provide a breadth of information regarding oro-pharyngeal exercise-based rehabilitative strategies for dysphagia but will be lacking an in-depth discussion into the robust nature of the literature reviewed. For this reason, it is predicted that this review will help define a more specific question for a systematic review in the future.

Due to time and funding limitations, source databases will be searched on one date before screening commences and updated once if the screening process takes more than 3 months. The date of these searches will be clearly recorded in future dissemination to ensure articles published after this date are considered in future reviews.

Further limitations include the exclusion of literature not published in English which increases the possibility of cultural bias.

### Dissemination

The results of this scoping review will be disseminated via publication in a peer-reviewed journal focused on the dissemination of dysphagia-related research and presentation at national/international conferences.

## Supplementary Information

**Additional file 1.** Preferred Reporting Items for Systematic reviews and Meta-Analyses extension for Scoping Reviews (PRISMA-ScR) Checklist.

**Additional file 2.** Draft search strategy.

## Data Availability

Data sharing is not applicable to this article as no datasets were generated or analysed during the current study.

## Abbreviations

ABI: Acquired brain injury
AMED: Allied and Complementary Medicine Database
CINAHL: Cumulative Index to Nursing and Allied Health Literature
CP: Cerebral palsy
CVA: Cerebrovascular accident
JBI: Joanna Briggs Institute
MeSH: Medical Subject Headings
NIHR: National Institute Health Research
PRISMA: Preferred Reporting Items for Systematic Reviews
RCSLT: Royal College of Speech and Language Therapists
SLT: Speech and language therapists
