# Supplementary material for: Dysphagia rehabilitation following acquired brain injury, including cerebral palsy, across the lifespan: a scoping review protocol
Source: Syst Rev. 2021 Dec 13;10:312. doi: 10.1186/s13643-021-01861-9 (PMC8667523; doi:10.1186/s13643-021-01861-9)
Supplement: Supplementary file 2 — Additional file 2. Draft search strategy. [file 13643_2021_1861_MOESM2_ESM.docx]

| **Level 1:** | **Rehabilitation** | exp Rehabilitation/ OR Stroke Rehabilitation/ OR rehabilitation.fs OR rehabilitat* |
| --- | --- | --- |
| **Level 2:** | **Dysphagia** | “Deglutition Disorders/” OR “deglutition disorder*” OR swallow* OR dysphagia |
| **Level 3:** | **Brain Injury** | “Brain Injuries/” OR “Brain Damage, Chronic/” OR “Brain Neoplasms/” OR Stroke/ OR “Cerebral Palsy/” OR infarc* OR embolism OR h?emorrhag* OR aneurysm* OR anoxi* OR hypoxi* OR isch?emi*OR thrombos* OR occlus* OR bleed* OR concuss* OR TBI OR ABI OR OR “brain neoplasm*” OR “brain cancer*” OR “brain tumo?r*” OR “intracranial neoplasm*” OR “cerebral palsy” OR “spastic diplegia” OR stroke OR “cerebrovascular accident*” OR “cerebral vascular accident*” OR “brain vascular accident*” OR CVA |
| **Level 4:** | **Paediatric** | Adolescent/ OR Child/ OR Infant/ OR Pediatrics/ OR Disabled Children/ OR p?ediatric* or child* or infant* or adolescen* OR toddler* OR school* OR schoolchild* OR teen* OR youth* OR “young person*” OR “young people*” OR juvenile* |

Additional file 2:

Draft search strategy.
